# Evaluation of the impact of the COVID-19 pandemic on health service utilization in China: A study using auto-regressive integrated moving average model

**DOI:** 10.3389/fpubh.2023.1114085

**Published:** 2023-04-06

**Authors:** Rixiang Xu, Lang Wu, Yulian Liu, Yaping Ye, Tingyu Mu, Caiming Xu, Huiling Yuan

**Affiliations:** ^1^School of Humanities and Management, Zhejiang Chinese Medical University, Hangzhou, China; ^2^School of Nursing, Zhejiang Chinese Medical University, Hangzhou, China; ^3^School of Law, Hangzhou City University, Hangzhou, China

**Keywords:** public emergencies, emergency management, health services access, forecast model, health policy

## Abstract

**Background:**

The outbreak of COVID-19 in early 2020 presented a major challenge to the healthcare system in China. This study aimed to quantitatively evaluate the impact of COVID-19 on health services utilization in China in 2020.

**Methods:**

Health service-related data for this study were extracted from the China Health Statistical Yearbook. The Auto-Regressive Integrated Moving Average model (ARIMA) was used to forecast the data for the year 2020 based on trends observed between 2010 and 2019. The differences between the actual 2020 values reported in the statistical yearbook and the forecast values from the ARIMA model were used to assess the impact of COVID-19 on health services utilization.

**Results:**

In 2020, the number of admissions and outpatient visits in China declined by 17.74 and 14.37%, respectively, compared to the ARIMA model’s forecast values. Notably, public hospitals experienced the largest decrease in outpatient visits and admissions, of 18.55 and 19.64%, respectively. Among all departments, the pediatrics department had the greatest decrease in outpatient visits (35.15%). Regarding geographical distribution, Beijing and Heilongjiang were the regions most affected by the decline in outpatient visits (29.96%) and admissions (43.20%) respectively.

**Conclusion:**

The study’s findings suggest that during the first year of the COVID-19 pandemic, one in seven outpatient services and one in six admissions were affected in China. Therefore, there is an urgent need to establish a green channel for seeking medical treatment without spatial and institutional barriers during epidemic prevention and control periods.

## 1. Introduction

At the close of 2019, coronavirus disease 2019 (COVID-19), a severe infectious disease, rapidly disseminated to numerous countries worldwide within a few months. The outbreak brought health systems, education, entertainment, commerce, tourism, and manufacturing industries globally to a near standstill (1). As of 17:00 on March 7, 2023, Beijing time, the novel coronavirus has undergone several rounds of mutations and has caused 758,390,564 infections and 6,859,093 deaths worldwide. In the early stages of the outbreak, limited information on the virus’ pathogenesis and mode of transmission resulted in high infection rates and direct mortality (2, 3). On January 30, 2020, the World Health Organization (WHO) declared that the COVID-19 a public health emergency of international concern. Subsequently, COVID-19 prevention and control measures, such as social distancing, personal protective equipment use, and self-isolation were significantly upgraded (4).

COVID-19 was first reported in Wuhan, and it had spread to all the 31 provinces of China by the end of January 2020 (5). To prevent the further spread of the pandemic, the provinces of mainland China quickly launched the highest level of response (6), including large-scale isolation of infected individuals or those at risk of infection, suspension of production and commercial activities in some areas, closure of some non-communicable disease hospitals and community hospitals, and restriction of non-essential social activities (7). Although these measures quickly reduced the number of infections at the social level to zero, they also generated spatial and institutional barriers that constrained public access to health services to some extent, especially during the pandemic. Moreover, the COVID-19 outbreak also disrupted other health resources, resulting in the closure of some non-emergency departments in China (8). Reduced access to health services, including essential health services, is one of the harmful manifestations of the prolonged COVID-19 epidemic and needs to be quantified to assess its objective impact. Several countries have recently reported quantitative data on the pandemic’s impact on health service utilization. For instance, during the first wave of the pandemic, health service utilization in the UK dropped by 70% (9). In Australia, manual therapy service utilization by private agencies decreased by approximately 7% during the first half of 2020 (10). However, there is a lack of national-level quantitative studies on this topic in China. Therefore, this study uses long-term data based on the Auto Regressive Integrated Moving Average model (ARIMA) to predict theoretical health service utilization data in 2020 if this outbreak had not occurred. The differences between the predicted and actual values in 2020 are then compared to quantify the impact of COVID-19 on health services utilization.

## 2. Methods

### 2.1. Data sources and study design

The China Health (Health and Family Planning) Statistical Yearbook is a comprehensive national-level health service statistics manual edited by the National Health (Health and Family Planning) Commission. Each edition of the yearbook presents China’s health-related data for the preceding year, encompassing data on health resource allocation, health expenditure, health service utilization, and population health-related indicators. Notably, the data covered 31 provinces in mainland China, excluding Hong Kong, Macau, and Taiwan, due to inconsistent statistical standards. In this study, we obtained data from the yearbook spanning the period 2010 and 2020. Target data from 2010 to 2019 were input into the ARIMA forecast model to predict the 2020 data in the absence of the outbreak. The data from the Statistical Yearbook for 2020 are after the impact of the pandemic. To roughly estimate the impact of the pandemic on health service utilization, we calculated the difference between the actual and forecast values for 2020. To clarify the characteristics of the impact, we conducted comparisons of data across different types of hospitals, 21 clinical departments, and 31 provinces. For simplicity, “province” here refers specifically to the original province (such as Hubei), municipality (such as Tianjin), and autonomous region (such as Xinjiang). The data were organized chronologically to establish a group of time series.

### 2.2. Auto-regressive integrated moving average model

The ARIMA model, was originally developed by Box and Jenkins as a forecasting tool for economic variables, is also known as the Box-Jenkins method (11). The first half of the statistical analysis of this study, a time series analysis was conducted to predict future values of the series. ARIMA is one of the most popular linear models for forecasting time series due to its ability to account for changing trends, periodic variations, and stochastic perturbations in time series. Given that the change in health service utilization is driven by multiple factors, the ARIMA model is considered to be the most appropriate model under existing conditions.

The ARIMA model is typically specified as a simple ARIMA (p, d, q) model, a seasonal ARIMA (P, D, Q) S model, and a seasonal-product ARIMA (p, d, q) (P, D, Q) S model, where p, d, q and P, D, Q are the continuous and seasonal order of autoregression, degree of difference, and order of moving average, respectively (12). As this study did not involve seasonal data, only the simple ARIMA model was utilized for the statistical analysis.

### 2.3. Models construction and selection

The ARIMA model is developed through four synergistic steps, which include time series stationary, model identification, parameter estimation, and diagnostic checking (13). Initially, ARIMA models necessitate a stationary time series. and the Augmented Dickey-Fuller (ADF) unit-root test can determine whether a time series is stationary. The parameters of the ARIMA model were estimated using autocorrelation function (ACF) plot and partial autocorrelation (PACF) plot, and the “auto.arima()” command in R software was employed to promptly identify the most suitable model. Finally, the Ljung-Box test was performed to confirm that temporal autocorrelation no longer existed in the model residuals (13, 14).

The statistical appropriateness and predictive accuracy of the selected models were assessed using Mean Absolute Percentage Error (MAPE), whereby lower values indicated a better fit of the data (15) (13). MAPE is represented by equation (1) below. ARIMA model selection and MAPE results for all time-series analyzes are elaborated in Supplementary Table S1.

(1) $$\mathrm{MAPE}=\frac{1}{n}\overset{n}{\underset{i=1}{\sum }}|\frac{e_{i}}{x_{i}}|$$

Where *n* is the number of time points, x_i_ is the actual value at time point i, and e_i_ is the difference between the actual and forecast values.

### 2.4. Data analysis

Microsoft Excel 2016 was used for data extraction and initial statistical analysis. ARIMA models were developed using the forecast package and tseries package in R software 4.1.2 to forecast 2020 values based on the existing time series (2010–2019). The output of this model included the forecast value and its 95% confidence interval (95%CI). The differences between the forecast values and the actual values in 2020 and the percentage changes (2) were derived to indicate the impact of COVID-19 on health service utilization.

(2) $$\mathrm{Percentage change}=\frac{\left(V_{\mathrm{forecast}}-V_{\mathrm{actual}}\right)\times 100}{V_{\mathrm{forecast}}}$$

Where V_forecast_ is the forecast value for 2020, V_actual_ is the actual value for 2020.

## 3. Results

### 3.1. Overall results and different types of hospitals

**Figure 1:**
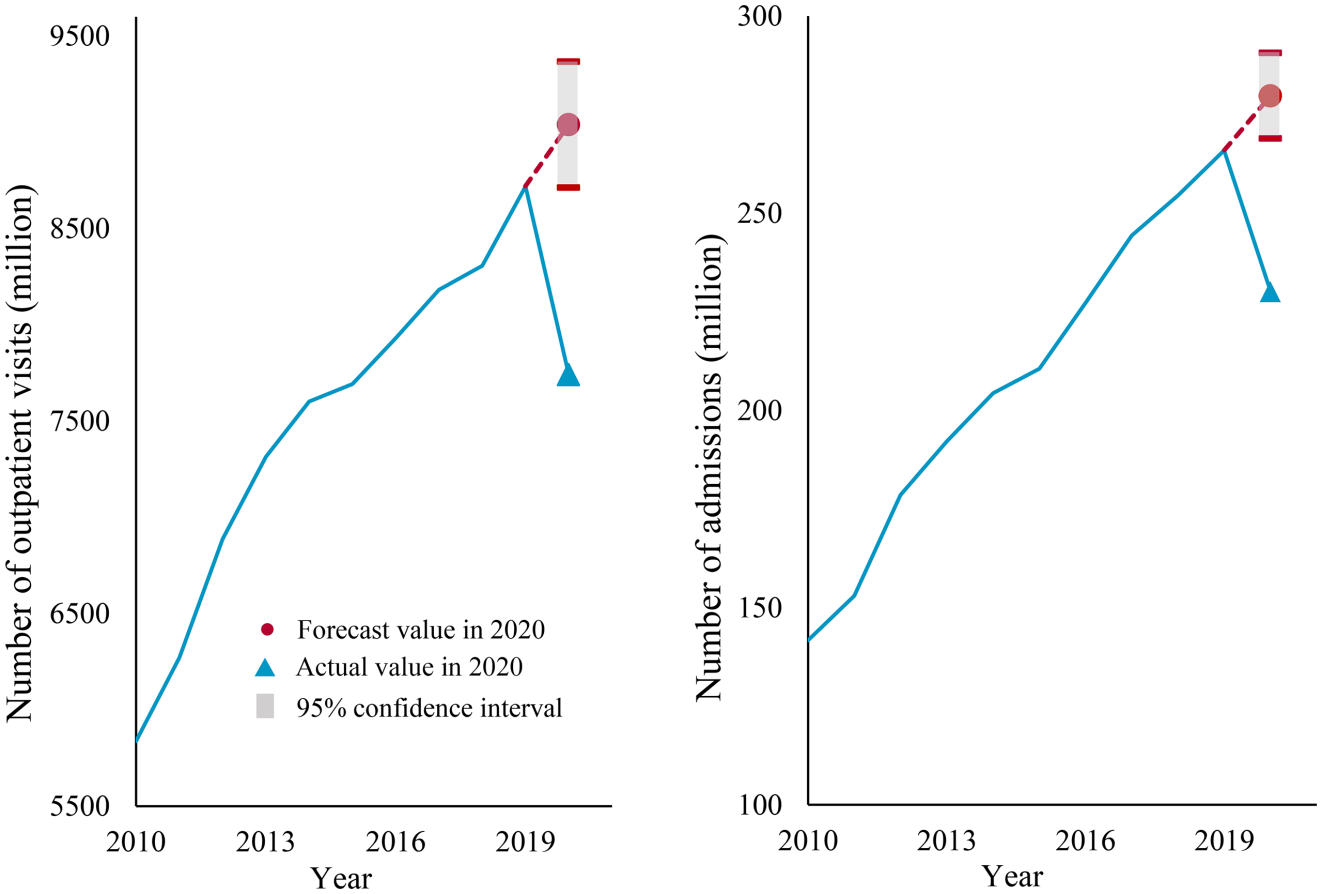
Actual and forecast values of overall health service utilization in 2020.

In 2020, the total number of outpatient visits and admissions in China were 7.74 billion and 2.30 billion, respectively. Between 2010 and 2019, there was an increase in the number of admissions and outpatient visits in all types of hospitals, regions, and almost all departments (except for the admissions in the prevention health department). Overall, the forecast values of admissions and inpatient visits in 2020 were 9.04 billion and 2.80 billion, respectively, representing a total decrease of 1.30 billion outpatient visits and 500 million admissions during the year, corresponding to percentage changes of 14.37 and 17.74%, respectively (Figure 1).

**Figure 2:**
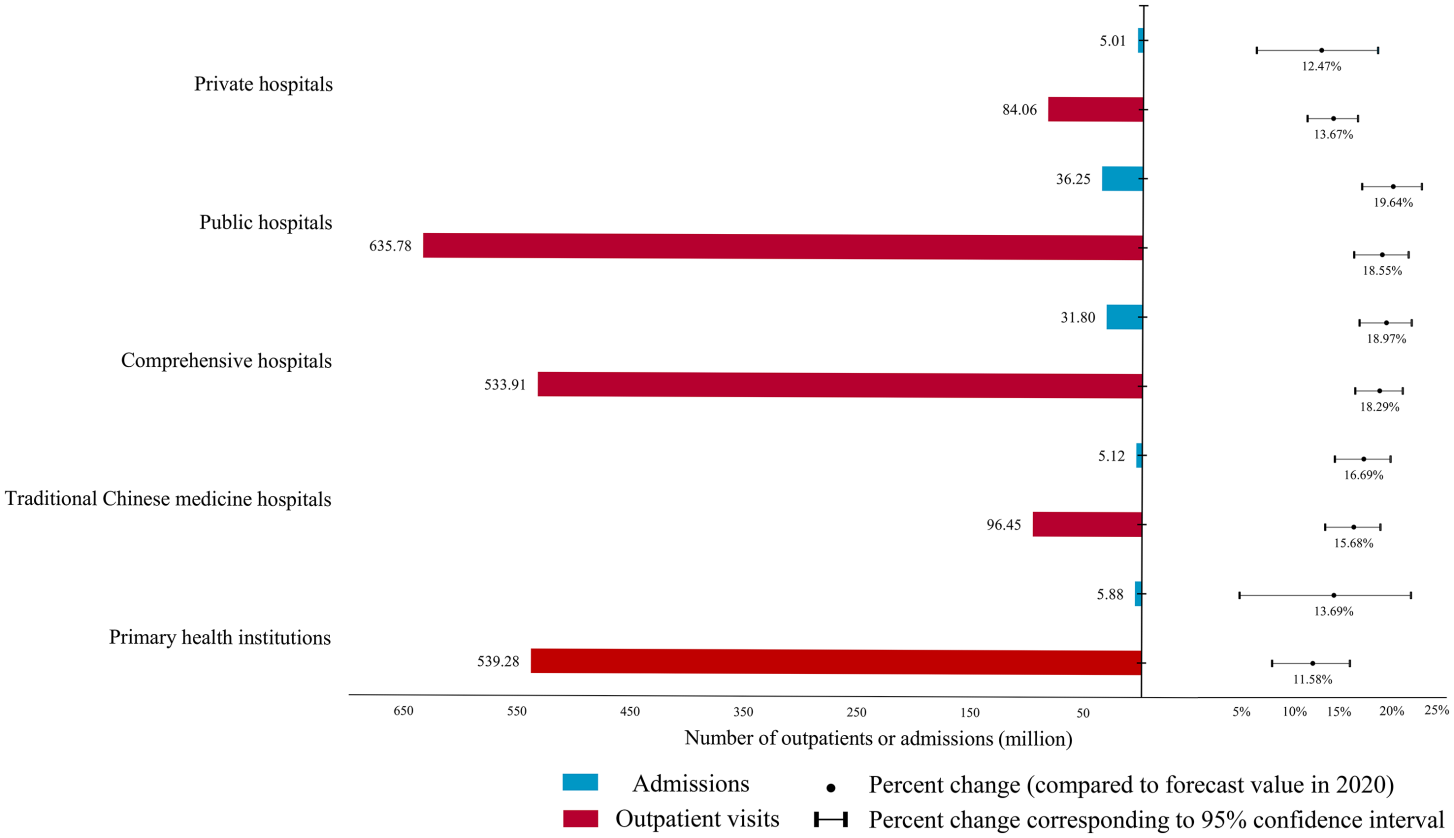
The difference between the forecast and actual health service utilization in different types of hospitals in 2020 and its percentage change.

In China, public hospitals remain the primary providers of health service and were the most impacted by the pandemic, with a percentage change of 18.55% (635.78 million) outpatient visits and 19.64% (36.25 million) admissions in 2020. Conversely, admissions in private hospitals and outpatient visits in primary hospitals were relatively less impacted, with percentage changes of 12.47% (539.28 million) and 11.58% (50,100), respectively. Traditional Chinese medicine (TCM) hospitals had smaller reductions in outpatient visits and admissions compared with comprehensive hospitals, with percentage changes of 15.68% versus 18.29 and 16.69% versus 18.97%, respectively (See Figure 2 for more results).

### 3.2. Results between different hospital departments

**Figure 3:**
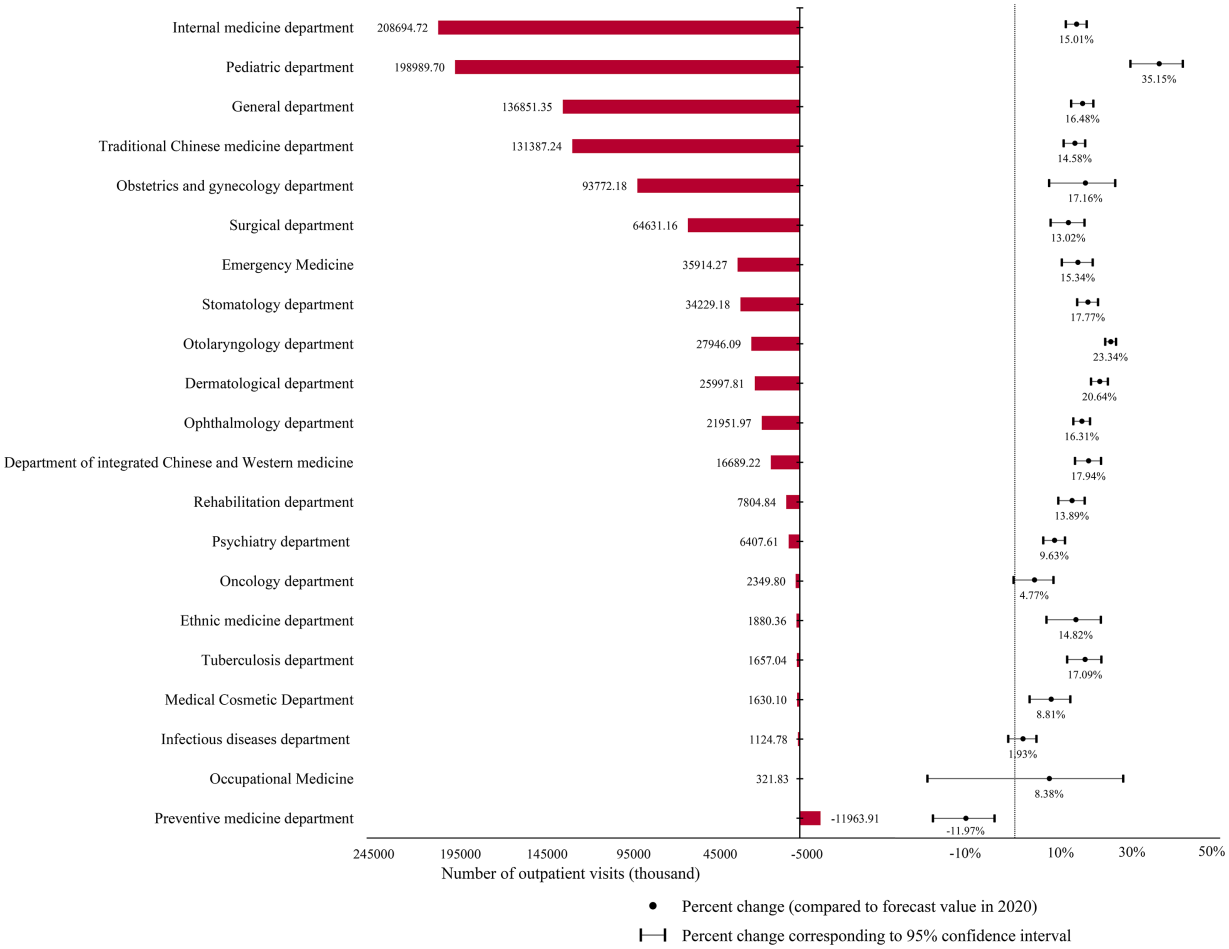
The difference between the predicted and actual values of outpatient visits in different hospital departments in 2020 and the percentage change in it.

Figure 3 displays the outpatient service utilization for different departments. Except for the preventive medicine department, which had a higher than predicted value of outpatient visits in 2020, all other departments had fewer numbers of outpatient visits by 321.83 thousand to 208.69 million in 2020. Among them, internal medicine, pediatrics, and general medicine departments had the largest reductions, of 208.69 million, 198.99 million, and 136.85 million, respectively. Pediatrics, otolaryngology, and dermatology were the top three departments based on percentage change, with reductions of 35.15, 23.34, and 20.64%, respectively. Conversely, the change rates in oncology, infectious diseases, and preventive health care were 4.77, 1.93%, and-11.97%, respectively, indicating that they were less affected by the pandemic.

**Figure 4:**
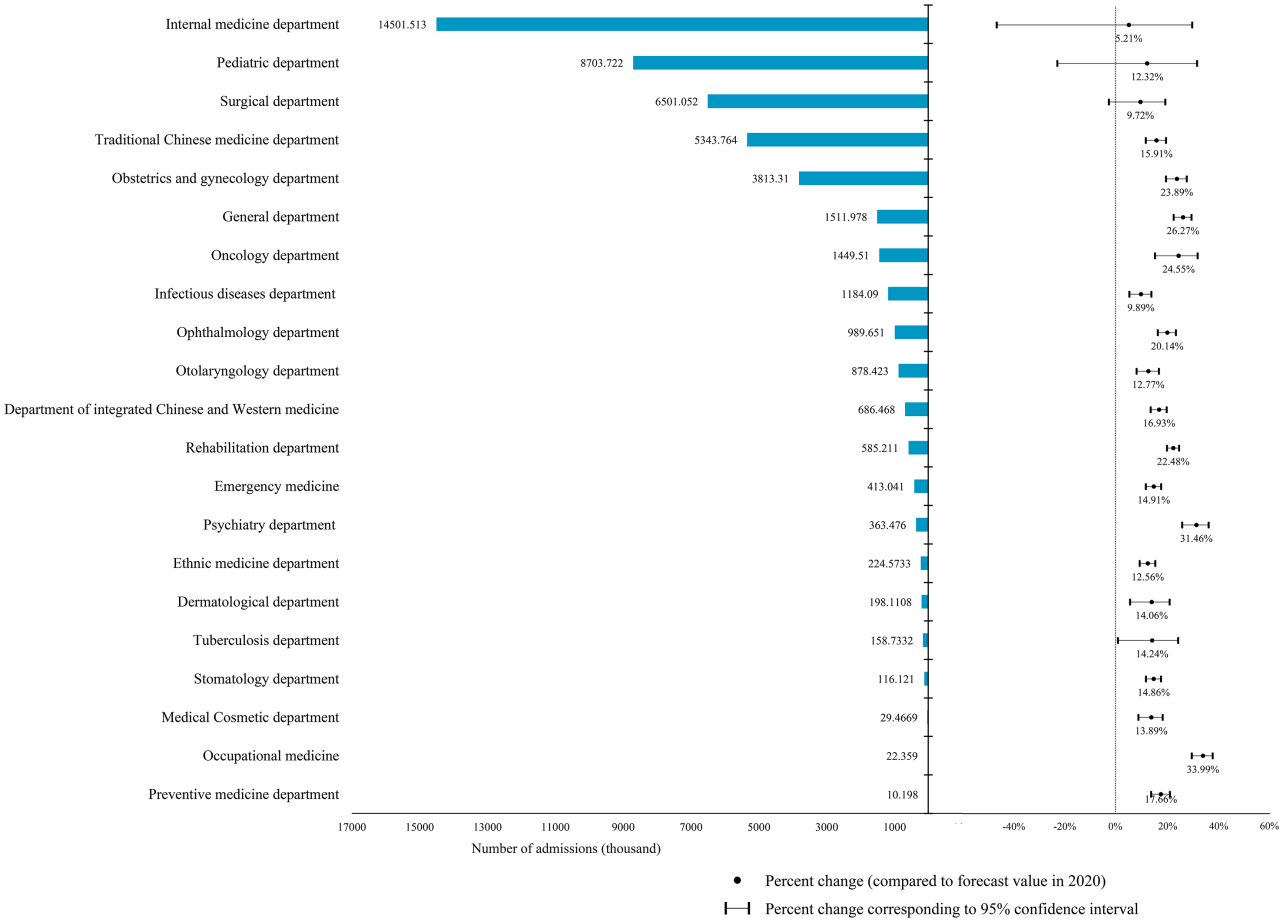
The difference between the predicted and actual values of admissions in different hospital departments in 2020 and the percentage change in it.

Figure 4 illustrates the impact of the pandemic on hospital admissions in different departments. The actual number of admissions in all departments was lower than predicted, with reductions ranging from 10.20 thousand to 14.50 million. Among them, internal medicine, pediatrics, and surgery were the three most affected departments, with reductions of 14.50 million, 8.70 million, and 6.50 million, respectively. However, the internal medicine and the surgery departments had the lowest percentage changes among those of all departments, at 5.21 and 9.72%, respectively. The top three departments with the highest percentage changes were occupational medicine, 33.99%; psychiatry, 31.46%; and general medicine, 26.27%. Additionally, the infectious diseases department, which is directly related to COVID-19, also had a reduction of 1.1841 million, with a percentage change of 9.89%.

### 3.3. Results between different regions

**Figure 5:**
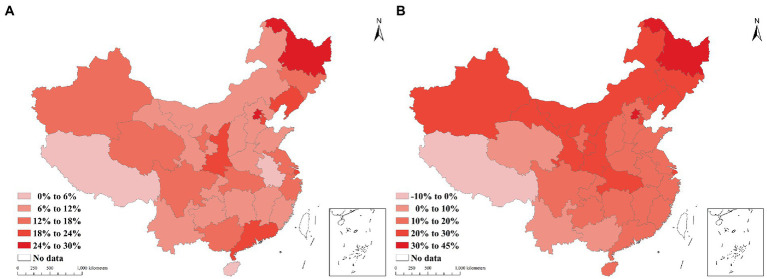
The percent change in health service utilization by province in 2020, **(A)** the percentage change in outpatient visits, **(B)** the percentage change in admissions.

The findings indicate that the actual number of outpatient visits in all provinces in 2020 was lower than estimated, with reductions ranging from 809.90 thousand to 197.59 million and a percentage change of 0.59 to 29.96% (Supplementary Table S2). Beijing, Heilongjiang, Tianjin, Guangdong, and Liaoning had percentage changes of more than one-fifth, at 29.96, 25.20, 22.23, 21.38, and 20.78%, respectively. In contrast, the impact of the epidemic on outpatient visits in Anhui, Hainan, and Tibet were relatively less, with percentage changes of 0.59, 2.03, and 4.47%, respectively (Figure 5A).

In Figure 5B, the number of admissions in the provinces decreased by between 106,710 and 4,226,794, except for Tibet, whose number of admissions increased by 25.49 thousand. The percentage changes in China revealed that the distribution characteristics decreased from north to south and from east to west. The number of admissions in Heilongjiang (43.20%) decreased by nearly half, and that in Beijing (37.69%) decreased by more than a third. In addition, the percentage changes in hospital admissions in Tianjin (27.04%), Jilin (26.90%), Hubei (29.18%), and Xinjiang (29.07%) were over a quarter. In contrast, the number of admissions in Tibet increased by 8.34%.

### 3.4. Results of model selection and MAPE

Supplementary Table S1 shows the selection of ARIMA models and MAPE values for each time series. The results indicate that the ARIMA (0,1,0) model is the most appropriate for the majority of health service utilization data sets, implying that only one differencing is required to render the time series stationary. Moreover, the MAPE values for the prediction models for total admission and outpatient service utilization are found to be 1.68 and 1.78%, respectively. Additionally, the MAPE ranges of the ARIMA prediction models for different types of hospitals, departments and regions are 1.36–3.66%, 0.76–10.47% and 0.95–10.38%, respectively. See Supplementary Table S1 for other details.

## 4. Discussions

The COVID-19 pandemic has wrought significant disruption to the normal social order worldwide in its first year. To our knowledge, this study represents the first attempt to investigate the impact of the first year of the pandemic on health service utilization in China, based on real-world data. The study reveals several key findings. First, the number of outpatient visits and admissions in China decreased by 14.37 and 17.34%, respectively, in 2020. Second, health service utilization was adversely impacted to varying degrees across virtually all types of hospitals, departments, and regions. Third, there was a reduction of more than a third outpatient visits in the pediatrics department and admissions in the occupational medicine department. Fourth, Beijing and Northeast China were among the regions whose health service utilization was most affected. These results provide quantitative evidence of the pandemic’s devastating social impact.

Comparing our findings to those reported in other countries can shed light on the global scope and patterns of pandemic-related disruptions in health service utilization. A growing body of literature has documented the negative effects of the COVID-19 pandemic on health services access and utilization in various settings. For example, a recent study from UK found that health service utilization decreased by 70% and respiratory system disease treatment services by 42% during the first wave of the pandemic. (9) Another study in Yemen reported a 10% reduction in surgery and a 4% decrease in medical consultation during the early phase of the pandemic in 2020 (16). Similar trends were observed in other countries, such as Armenia (17), Iran (18), and Italy (19). However, the magnitude and duration of the declines varied across countries and healthcare sectors, reflecting differences in the pandemic’s severity, public health response, healthcare system capacity, and patient behavior. By highlighting the similarities and differences between our results and those of other countries, we can better understand the multifaceted challenges and opportunities for healthcare delivery and policy during and beyond the pandemic.

In China, the impact of the COVID-19 epidemic on the utilization of health services are multifaceted and can be attributed to two dimensions. First, the direct impact of the epidemic. At the onset of the outbreak, the lack of knowledge about the transmission and virulence of the novel coronavirus resulted in the destruction of the local health service system. Consequently, the government dispatched a large number of health staff from hospitals across the country to assist areas with uncontrolled outbreaks (20). During the normalization stage, there were small and controllable outbreaks in some cities. Doctors in hospitals were tasked with collecting sample from residents for nucleic acid analysis. Moreover, the high risk of infection also led to doctors being isolated at home or in designated facilities thereby reducing the provision of health services in the short term. Second, epidemic prevention and control policies have also contributed to the challenges in accessing health services. China is one of the few countries in the world that implemented a “zero-COVID” policy (referring to the government’s efforts to stop the spread of the epidemic so that there are no COVID-19 patients at the social level) (21). which restricts non-essential outdoor activities for residents in endemic areas for at least 2 weeks to contain the spread of the epidemic (22). Meanwhile, primary health institutions were closed, and only one outpatient department handling patients with fever was left to serve COVID-19 patients and suspects (23). There are even large hospitals that temporarily closed outpatient services to prevent nosocomial infections. These policies created spatial barriers to local patients accessing health services. To address the spatial inaccessibility of health services in endemic areas, some hospitals have offered telemedicine services to patients (24, 25). Nevertheless, online medical services lack objective diagnostic evidence (such as biochemical tests), limiting the medical assistance that can be provided to patients.

The impact of the COVID-19 pandemic on health services utilization varies across hospital departments and regions. The pediatrics department experienced the greatest decline in outpatient visits, losing over one-third of its patients in 2020, ranking first among all departments. Pediatrics is one of the busiest medical specialties in China, and previous studies have reported that pediatricians work more intensively than non-pediatricians (26). Consequently, the absence of pediatricians due to the epidemic had a far greater impact on outpatient services utilization than the absence of other specialists. Moreover, fears of nosocomial infections and complicated procedures (e.g., timely negative nucleic acid test report of the coronavirus as a pass to enter the hospital) during the pandemic made parents to delay hospital visits for their children with non-emergency conditions. However, it is worth mentioning that the number of outpatient visits only increased in the preventive health department. One possible explanation is that the COVID-19 outbreak promoted the demand for services such as infectious disease prevention, vaccination, and health education in this department.

Beijing, as the capital of China, has an abundance of health resources and attracts countless patients to seek medical treatment annually (27). However, travel restrictions between different regions during the early stages of the pandemic resulted in fewer out-of-town patients migrating to Beijing for medical treatment, making it one of the cities most affected by the pandemic’s impact on health service utilization. Additionally, travel restrictions during the Spring Festival prevented migrant workers from leaving their hometowns, leading to a relatively low decline in health services in labor-exporting provinces such as Anhui Province was relatively low. The differences in the pandemic’s impact on health service utilization across regions may be related to local epidemic prevention and control measures. For instance, Tibet, which had only one COVID-19 case in 2020, had a larger than predicted number of admissions (8.34%), while Heilongjiang, which had more than 1,000 cumulative cases in the same year, experienced the greatest decline in admissions (43.20%) (28).

The health service utilizations (unoccurring health service demands) affected by COVID-19 are objectively divided into necessary and non-necessary. For necessary health services, especially emergency services, need to be paid attention to and addressed. At the policy level, several recommendations can be made to address this issue. Firstly, governments should establish a system of barrier-free access to health services, including both institutional and spatial accessibility, in preparation for potential pandemics. Secondly, doctors and nurses, especially pediatricians, should not be deployed to participate in sample collection for nucleic acid analysis unless necessary, so that they can focus on their primary healthcare duties. Thirdly, telemedicine should be taken full advantage of to improve the spatial accessibility of health services. Lastly, primary healthcare institutions should not be closed during epidemics, as this can cause further strain on the healthcare system.

Several limitations must be considered when interpreting our findings. First, China launched healthcare reforms in 2009, and since then has issued a series of health policies (29). These policies may have influenced the consumption and provision of health services, and thus may have affected the accuracy of our ARIMA model predictions. Second, the interpretation of our results is based on the reports of previous studies and empirical reasoning; there may be some bias, and care should be taken when interpreting our findings. Third, our study did not consider the impact of health service utilization in Fangcang hospitals, where COVID-19 patients were centrally isolated and treated. Fourth, the accuracy of the predicted results is related to the model’s MAPE value, and researchers referring to our findings should take these into consideration. Fifth, we relied mainly on official data sources that may not fully reflect the real-world situation. Lastly, due to the time lag of official data publication, this study only examined the impact of the 2020 pandemic on health service utilization.

Based on the findings and limitations of this study, we suggest several directions for future research. First, future research should examine the effects of COVID-19 on the quality of care and health outcomes of patients who utilized health services during the pandemic. This would provide a comprehensive assessment of how the pandemic impacted the quality and effectiveness of health service delivery and whether it created any gaps or disparities in care. Second, future research should investigate the determinants and patterns of health service utilization among specific populations, such as elderly people or people with chronic conditions. These populations may have distinct needs and preferences for health services and may face different barriers and risks during the pandemic. This would enhance our understanding of their experiences and expectations for health services and inform the development of tailored interventions to improve their access and satisfaction.

## 5. Conclusion

The study quantifies the impact of COVID-19 on health services utilization in China. In 2020, the actual utilization of admissions and outpatient services decreased by about one-sixth and one-seventh, respectively, as compared to the values predicted by the ARIMA model. The reasons for the impact on health service utilization are multifaceted, encompassing the direct effects of COVID-19 and its prevention and control policies. In the future, governments must establish a mechanism to enhance access to health resources for patients in need of health services (especially emergencies) during infectious disease pandemics.

## Data availability statement

The original contributions presented in the study are included in the article/Supplementary material, further inquiries can be directed to the corresponding authors.

## Author contributions

RX: literature search, data collection and analysis, and manuscript preparation. CX: study design and guidance and financial support. TM: data collection and analysis. YL: data presentation and manuscript revision. YY: literature search and manuscript preparation. LW: figure making, data collection, and analysis. All authors contributed to the article and approved the submitted version.

## Funding

This research was supported by the Zhejiang Provincial Science and Technology Program of Traditional Chinese Medicine (grant number: 2023ZF009), and research project of Zhejiang Federation of Social Sciences (grant number: 2021 N14).

## Conflict of interest

The authors declare that the research was conducted in the absence of any commercial or financial relationships that could be construed as a potential conflict of interest.

## Publisher’s note

All claims expressed in this article are solely those of the authors and do not necessarily represent those of their affiliated organizations, or those of the publisher, the editors and the reviewers. Any product that may be evaluated in this article, or claim that may be made by its manufacturer, is not guaranteed or endorsed by the publisher.

## References

[1] Mbunge E. Integrating emerging technologies into covid-19 contact tracing: opportunities, challenges and pitfalls. Diabetes Metab Syndr Clin Res Rev. (2020) 14:1631–6. doi: 10.1016/j.dsx.2020.08.029, PMID: 32892060 PMC7833487

[2] Wiersinga WJ Rhodes A Cheng AC Peacock SJ Prescott HC. Pathophysiology, transmission, diagnosis, and treatment of coronavirus disease 2019 (covid-19): a review. JAMA. (2020) 324:782–93. doi: 10.1001/jama.2020.12839 32648899

[3] Huang C Wang Y Li X. Clinical features of patients infected with 2019 novel coronavirus in Wuhan, China. Lancet. (2020) 395:497–506. doi: 10.1016/S0140-6736(20)30183-5, PMID: 31986264 PMC7159299

[4] Chang AY Cullen MR Harrington RA Barry M. The impact of novel coronavirus covid-19 on noncommunicable disease patients and health systems: a review. J Intern Med. (2021) 289:450–62. doi: 10.1111/joim.13184, PMID: 33020988 PMC7675448

[5] Xing C Zhang R. Covid-19 in China: responses, challenges and implications for the health system. Healthcare (Basel). (2021) 9:9. doi: 10.3390/healthcare9010082, PMID: 33466996 PMC7830573

[6] Zhang P Gao J. Evaluation of China’s public health system response to covid-19. J Glob Health. (2021) 11:5004. doi: 10.7189/jogh.11.05004, PMID: 33643637 PMC7897426

[7] Zhang S Wang Z Chang R Wang H Xu C Yu X. Covid-19 containment: China provides important lessons for global response. Front Med. (2020) 14:215–9. doi: 10.1007/s11684-020-0766-9, PMID: 32212059 PMC7095399

[8] Yu X Li N. Understanding the beginning of a pandemic: China’s response to the emergence of covid-19. J Infect Public Health. (2021) 14:347–52. doi: 10.1016/j.jiph.2020.12.024, PMID: 33618279 PMC7836925

[9] Howarth A Munro M Theodorou A Mills PR. Trends in healthcare utilisation during covid-19: a longitudinal study from the UK. BMJ Open. (2021) 11:e48151. doi: 10.1136/bmjopen-2020-048151, PMID: 34330859 PMC8327639

[10] Lystad RP Brown BT Swain MS Engel RM. Impact of the covid-19 pandemic on manual therapy service utilization within the Australian private healthcare setting. Healthcare (Basel). (2020) 8:558. doi: 10.3390/healthcare8040558, PMID: 33322226 PMC7764415

[11] Box GEP Jenkins GM Reinsel GC Ljung GM. Control, Time Series Analysis Forecasting. Hoboken, New Jersey: John Wiley and Sons (2015).

[12] Wang M Pan J Li X Li M Liu Z Zhao Q. Arima and Arima-ernn models for prediction of pertussis incidence in mainland China from 2004 to 2021. BMC Public Health. (2022) 22:1447. doi: 10.1186/s12889-022-13872-9, PMID: 35906580 PMC9338508

[13] Zheng A Fang Q Zhu Y Jiang C Jin F Wang X. An application of Arima model for predicting total health expenditure in China from 1978-2022. J Glob Health. (2020) 10:10803. doi: 10.7189/jogh.10.010803, PMID: 32257167 PMC7101215

[14] Wang YW Shen ZZ Jiang Y. Comparison of Arima and gm (1, 1) models for prediction of hepatitis b in China. PLoS One. (2018) 13:e201987. doi: 10.1371/journal.pone.0201987, PMID: 30180159 PMC6122800

[15] Ceylan Z. Estimation of covid-19 prevalence in Italy, Spain, and France. Sci Total Environ. (2020) 729:138817. doi: 10.1016/j.scitotenv.2020.138817, PMID: 32360907 PMC7175852

[16] Kotiso M Qirbi N Al-Shabi K Vuolo E Al-Waleedi A Naiene J. Impact of the covid-19 pandemic on the utilisation of health services at public hospitals in Yemen: a retrospective comparative study. BMJ Open. (2022) 12:e47868. doi: 10.1136/bmjopen-2020-047868, PMID: 34980605 PMC8724586

[17] Abdoulaye MB Oumarou B Moussa H Anya BM Didier T Nsiari-muzeyi BJ. Impact de la pandémie de la covid-19 sur l’utilisation des services de santé dans la ville de niamey: une analyse dans 17 formations sanitaires de janvier à juin 2020. Pan Afr Med J. (2021) 31:159. doi: 10.11604/pamj.2021.39.159.28282 PMC8434778 34539956

[18] Rezapour R Dorosti AA Farahbakhsh M Azami-aghdash S Iranzad I. The impact of the covid-19 pandemic on primary health care utilization: an experience from Iran. BMC Health Serv Res. (2022) 22:404. doi: 10.1186/s12913-022-07753-5, PMID: 35346175 PMC8960210

[19] Di Girolamo C Gnavi R Landriscina T Forni S Falcone M Calandrini E. Indirect impact of the covid-19 pandemic and its containment measures on social inequalities in hospital utilisation in Italy. J Epidemiol Community Health. (2022) 76:707–15. doi: 10.1136/jech-2021-218452, PMID: 35552241

[20] Xie L Yang H Zheng X Wu Y Lin X Shen Z. Medical resources and coronavirus disease (covid-19) mortality rate: evidence and implications from Hubei province in China. PLoS One. (2021) 16:e244867. doi: 10.1371/journal.pone.0244867, PMID: 33449940 PMC7810342

[21] Chen JM Chen YQ. China can prepare to end its zero-covid policy. Nat Med. (2022) 28:1104–5. doi: 10.1038/s41591-022-01794-3, PMID: 35383312

[22] Zuo Y Zhang M Han J Chen KW Ren Z. Residents' physical activities in home isolation and its relationship with health values and well-being: a cross-sectional survey during the covid-19 social quarantine. Healthcare (Basel). (2021) 9:795. doi: 10.3390/healthcare9070795, PMID: 34202912 PMC8307814

[23] Jinwei H Dongdong J Quan W Zongfu M. Construction of primary health service system in Wuhan after the epidemic of covid-19: from the perspective of stakeholders. Chin J Health Policy. (2020) 13:15–21. doi: 10.3969/j.issn.1674-2982.2020.09.003

[24] Hong Z Li N Li D Li J Li B Xiong W. Telemedicine during the covid-19 pandemic: experiences from western China. J Med Internet Res. (2020) 22:e19577. doi: 10.2196/19577, PMID: 32349962 PMC7212818

[25] Huang M Wang J Nicholas S Maitland E Guo Z. Development, status quo, and challenges to China’s health informatization during covid-19: evaluation and recommendations. J Med Internet Res. (2021) 23:e27345. doi: 10.2196/27345, PMID: 34061761 PMC8213061

[26] Ting G Liang Z Wei L. Researches on the path to pediatricians' sustainable development from the perspective of supply-side. Chin Health Serv Manag. (2021) 38:94–9. doi: 10.3969/j.issn.1004-4663.2021.02.004

[27] Guo Q Luo K Hu R. The spatial correlations of health resource agglomeration capacities and their influencing factors: evidence from China. Int J Environ Res Public Health. (2020) 17:17. doi: 10.3390/ijerph17228705, PMID: 33238597 PMC7700579

[28] Health Commission of Tibet Autonomous Region. The Outbreak of COVID-19 in the Tibet Autonomous Region on January 1. China: Health Commission of Tibet Autonomous Region (2022).

[29] Xu R Li S Lv X Xie X. Prices, availability, and affordability of national essential medicines in public primary hospitals: a cross-sectional survey in poverty-stricken rural areas in China. Int J Health Plann Manag. (2020) 35:545–57. doi: 10.1002/hpm.2963, PMID: 31736154

