# Supplementary material for: Evaluation of the impact of the COVID-19 pandemic on health service utilization in China: A study using auto-regressive integrated moving average model
Source: Front Public Health. 2023 Apr 6;11:1114085. doi: 10.3389/fpubh.2023.1114085 (PMC10115989; doi:10.3389/fpubh.2023.1114085)
Supplement: Supplementary file 1 [file Table_1.docx]

Table S1. Mean Absolute Percentage Error (MAPE) and ARIMA model selection for each time series

| **Groups** | | **Outpatient visits** | |  | **Admissions** | |
| --- | --- | --- | --- | --- | --- | --- |
|  |  | MAPE (%) | ARIMA Model (p, d, q) |  | MAPE (%) | ARIMA Model (p, d, q) |
| **Overall** | | 1.63 | 0,1,0 |  | 1.78 | 0,1,0 |
| Hospitals | Primary health institutions | 1.74 | 0,2,0 |  | 3.66 | 0,1,0 |
|  | Traditional Chinese medicine hospitals | 1.62 | 0,1,0 |  | 1.50 | 0,1,0 |
|  | Comprehensive hospitals | 1.40 | 0,1,0 |  | 1.36 | 0,1,0 |
|  | Public hospitals | 1.62 | 0,1,0 |  | 1.52 | 0,1,0 |
|  | Private hospitals | 1.61 | 0,1,0 |  | 3.53 | 0,1,0 |
| Departments | Preventive medicine department | 2.30 | 0,1,0 |  | 9.82 | 0,1,0 |
|  | General department | 1.43 | 0,1,0 |  | 4.01 | 0,0,0 |
|  | Internal medicine department | 1.35 | 0,1,0 |  | 2.03 | 0,1,0 |
|  | Surgical department | 1.76 | 0,1,0 |  | 2.20 | 0,1,0 |
|  | Pediatric department | 3.39 | 0,1,0 |  | 2.46 | 0,1,0 |
|  | Obstetrics and gynecology department | 3.74 | 0,1,0 |  | 5.02 | 0,1,0 |
|  | Ophthalmology department | 0.87 | 0,1,0 |  | 2.03 | 0,1,0 |
|  | Otolaryngology department | 0.76 | 0,1,0 |  | 1.58 | 0,1,0 |
|  | Stomatology department | 1.43 | 0,2,0 |  | 1.44 | 0,2,0 |
|  | Dermatological department | 1.27 | 0,1,0 |  | 2.45 | 0,1,0 |
|  | Medical Cosmetic Department | 3.40 | 0,2,0 |  | 7.70 | 0,1,0 |
|  | Psychiatry department | 1.43 | 0,2,0 |  | 2.85 | 0,2,0 |
|  | Infectious diseases department | 1.35 | 0,1,0 |  | 3.18 | 0,1,0 |
|  | Tuberculosis department | 2.05 | 0,1,0 |  | 2.36 | 0,1,0 |
|  | Oncology department | 3.51 | 0,1,0 |  | 2.26 | 0,2,0 |
|  | Emergency Medicine | 1.74 | 0,2,0 |  | 2.20 | 0,1,0 |
|  | Rehabilitation department | 1.62 | 0,1,0 |  | 3.85 | 0,1,0 |
|  | Occupational Medicine | 9.84 | 0,1,0 |  | 10.47 | 0,1,0 |
|  | Traditional Chinese medicine department | 1.54 | 0,1,0 |  | 1.80 | 0,1,0 |
|  | Ethnic medicine department | 4.19 | 0,1,0 |  | 5.72 | 0,1,0 |
|  | Department of integrated Chinese and Western medicine | 2.40 | 0,1,0 |  | 2.62 | 0,1,0 |
| Provinces | Beijing | 2.84 | 0,1,0 |  | 1.88 | 0,1,0 |
|  | Tianjin | 1.80 | 0,2,0 |  | 2.81 | 0,1,0 |
|  | Hebei | 1.46 | 0,2,0 |  | 2.58 | 0,1,0 |
|  | Shanxi | 3.01 | 0,1,0 |  | 3.26 | 0,1,0 |
|  | Inner Mongolia | 1.33 | 0,2,0 |  | 3.88 | 0,1,0 |
|  | Liaoning | 2.17 | 0,1,0 |  | 3.17 | 0,1,0 |
|  | Jilin | 2.21 | 0,2,0 |  | 2.26 | 0,1,0 |
|  | Heilongjiang | 2.94 | 0,0,1 |  | 3.60 | 0,1,0 |
|  | Shanghai | 1.04 | 0,2,0 |  | 1.79 | 0,1,0 |
|  | Jiangsu | 1.43 | 0,2,0 |  | 2.85 | 0,2,0 |
|  | Zhejiang | 1.63 | 0,1,0 |  | 1.32 | 0,1,0 |
|  | Anhui | 3.49 | 0,1,0 |  | 2.55 | 0,1,0 |
|  | Fujian | 1.65 | 0,1,0 |  | 2.86 | 0,1,0 |
|  | Jiangxi | 3.29 | 0,1,0 |  | 3.14 | 0,1,0 |
|  | Shandong | 2.61 | 0,1,0 |  | 3.32 | 0,1,0 |
|  | Henan | 2.17 | 0,2,0 |  | 2.50 | 0,1,0 |
|  | Hubei | 1.46 | 0,2,0 |  | 2.58 | 0,1,0 |
|  | Hunan | 1.25 | 0,1,0 |  | 1.48 | 0,2,0 |
|  | Guangdong | 1.92 | 0,1,0 |  | 1.27 | 0,1,0 |
|  | Guangxi | 2.63 | 0,1,0 |  | 4.68 | 0,1,0 |
|  | Hainan | 1.87 | 0,2,0 |  | 0.95 | 0,1,0 |
|  | Chongqing | 1.78 | 0,1,0 |  | 2.11 | 0,1,0 |
|  | Sichuan | 2.12 | 0,1,0 |  | 3.53 | 0,1,0 |
|  | Guizhou | 1.62 | 0,1,0 |  | 5.40 | 0,1,0 |
|  | Yunnan | 2.16 | 0,1,0 |  | 2.44 | 0,1,0 |
|  | Tibet | 4.52 | 0,1,0 |  | 10.38 | 0,1,0 |
|  | Shaanxi | 2.16 | 0,1,0 |  | 2.23 | 0,1,0 |
|  | Gansu | 2.80 | 0,2,0 |  | 3.20 | 0,1,0 |
|  | Qinghai | 2.24 | 0,1,0 |  | 3.42 | 0,1,0 |
|  | Ningxia | 1.49 | 0,1,0 |  | 2.94 | 0,1,0 |
|  | Xinjiang | 3.22 | 0,1,0 |  | 2.55 | 0,1,0 |
