# Supplementary material for: Evaluation of the impact of the COVID-19 pandemic on health service utilization in China: A study using auto-regressive integrated moving average model
Source: Front Public Health. 2023 Apr 6;11:1114085. doi: 10.3389/fpubh.2023.1114085 (PMC10115989; doi:10.3389/fpubh.2023.1114085)
Supplement: Supplementary file 2 [file Table_2.docx]

Table S2. Changes in the utilization of outpatient and inpatient services in 31 provinces in 2020

| Province | Outpatient visits (million) | | | |  | Admissions (million) | | | |
| --- | --- | --- | --- | --- | --- | --- | --- | --- | --- |
|  | Forecast value | Actual value | Difference | Percent change |  | Forecast value | Actual value | Difference | Percent change |
| Beijing | 260.25 | 182.29 | 77.96 | 29.96% |  | 4.07 | 2.54 | 1.53 | 37.69% |
| Tianjin | 125.79 | 97.83 | 27.96 | 22.23% |  | 1.77 | 1.29 | 0.48 | 27.04% |
| Hebei | 433.19 | 381.80 | 51.39 | 11.86% |  | 12.43 | 10.31 | 2.12 | 17.05% |
| Shanxi | 131.46 | 123.00 | 8.46 | 6.43% |  | 5.25 | 4.27 | 0.98 | 18.58% |
| Inner Mongolia | 108.54 | 96.12 | 12.42 | 11.44% |  | 3.79 | 2.94 | 0.85 | 22.30% |
| Liaoning | 205.74 | 162.99 | 42.75 | 20.78% |  | 7.37 | 5.76 | 1.61 | 21.90% |
| Jilin | 110.43 | 92.79 | 17.64 | 15.98% |  | 4.19 | 3.07 | 1.12 | 26.90% |
| Heilongjiang | 113.64 | 85.00 | 28.64 | 25.20% |  | 6.3 | 3.58 | 2.72 | 43.20% |
| Shanghai | 281.03 | 225.64 | 55.39 | 19.71% |  | 4.8 | 3.75 | 1.05 | 21.79% |
| Jiangsu | 643.06 | 533.56 | 109.50 | 17.03% |  | 16.15 | 13.57 | 2.58 | 16.03% |
| Zhejiang | 716.92 | 605.00 | 111.92 | 15.61% |  | 11.72 | 9.65 | 2.07 | 17.65% |
| Anhui | 348.12 | 346.07 | 2.05 | 0.59% |  | 10.9 | 9.5 | 1.4 | 12.81% |
| Fujian | 258.43 | 240.45 | 17.98 | 6.96% |  | 6.32 | 5.31 | 1.01 | 15.95% |
| Jiangxi | 245.16 | 219.95 | 25.21 | 10.28% |  | 9.27 | 8.07 | 1.2 | 12.99% |
| Shandong | 696.24 | 613.29 | 82.95 | 11.91% |  | 19.43 | 16.62 | 2.81 | 14.48% |
| Henan | 634.98 | 573.65 | 61.33 | 9.66% |  | 21.32 | 18.29 | 3.03 | 14.20% |
| Hubei | 356.16 | 294.56 | 61.60 | 17.29% |  | 14.49 | 10.26 | 4.23 | 29.18% |
| Hunan | 289.12 | 267.23 | 21.89 | 7.57% |  | 16.95 | 14.87 | 2.08 | 12.29% |
| Guangdong | 923.99 | 726.40 | 197.59 | 21.38% |  | 19.04 | 15.64 | 3.4 | 17.83% |
| Guangxi | 268.59 | 231.82 | 36.77 | 13.69% |  | 10.97 | 9.98 | 0.99 | 9.01% |
| Hainan | 54.27 | 53.17 | 1.10 | 2.03% |  | 1.35 | 1.16 | 0.19 | 14.30% |
| Chongqing | 182.07 | 170.28 | 11.79 | 6.47% |  | 7.99 | 6.76 | 1.23 | 15.39% |
| Sichuan | 582.32 | 512.28 | 70.04 | 12.03% |  | 20.84 | 17.56 | 3.28 | 15.73% |
| Guizhou | 183.91 | 162.09 | 21.82 | 11.86% |  | 9.09 | 7.81 | 1.28 | 14.04% |
| Yunnan | 294.26 | 269.83 | 24.43 | 8.30% |  | 10.7 | 9.7 | 1 | 9.32% |
| Tibet | 17.09 | 16.28 | 0.81 | 4.74% |  | 0.31 | 0.33 | -0.02 | -8.34% |
| Shaanxi | 216.29 | 176.64 | 39.65 | 18.33% |  | 8.69 | 6.76 | 1.93 | 22.27% |
| Gansu | 121.32 | 110.44 | 10.88 | 8.97% |  | 5.54 | 4.31 | 1.23 | 22.15% |
| Qinghai | 27.46 | 24.00 | 3.46 | 12.60% |  | 1.12 | 1.01 | 0.11 | 9.56% |
| Ningxia | 45.55 | 39.63 | 5.92 | 12.99% |  | 1.3 | 1.07 | 0.23 | 17.56% |
| Xinjiang | 125.42 | 107.00 | 18.42 | 14.69% |  | 6.16 | 4.37 | 1.79 | 29.07% |
